# The role of CYP2C9*2, CYP2C9*3 and *VKORC1-*1639 variants on the susceptibility of upper gastrointestinal bleeding: A full case-control study

**DOI:** 10.3389/jpps.2023.11136

**Published:** 2023-01-30

**Authors:** Marcela Forgerini, Gustavo Urbano, Tales Rubens De Nadai, Sabrina Setembre Batah, Alexandre Todorovic Fabro, Patrícia De Carvalho Mastroianni

**Affiliations:** ^1^ Department of Drugs and Medicines, School of Pharmaceutical Sciences, São Paulo State University (UNESP), Araraquara, São Paulo, Brazil; ^2^ Department of Surgery, School of Medicine, University of São Paulo (USP), Ribeirão Preto, São Paulo, Brazil; ^3^ Department of Public Health, Bauru School of Dentistry, University of São Paulo (USP), Bauru, São Paulo, Brazil; ^4^ Department of Pathology and Legal Medicine, Ribeirão Preto Medical School, University of São Paulo, Ribeirão Preto, São Paulo, Brazil

**Keywords:** cytochrome P-450 CYP2C9, pharmacogenomic variants, platelet aggregation inhibitors, non-steroidal anti-inflammatory agents, vitamin K epoxide reductases

## Abstract

**Purpose:** To investigate whether interindividual variability in the *CYP2C9* (*2 and *3 alleles) and *VKORC1* (rs9923231) genes is associated with increased risk of upper gastrointestinal bleeding (UGIB) in users of non-steroidal anti-inflammatory drugs (NSAIDs) or low-dose aspirin (LDA).

**Methods:** A full case-control study including 200 cases of patients diagnosed with UGIB and 706 controls was conducted in a Brazilian hospital complex. To perform an analysis of NSAIDs dose-effect, the defined daily dose (DDD) for NSAIDs was calculated in the 7-day etiologic window preceding the data index. Three categories of DDD, considering the genotypes of the genetic variants, were established: non-users of NSAIDs (DDD = 0), DDD ≤0.5, and DDD >0.5. Genetic variants and LDA or NSAIDs use synergism was estimated through Synergism Index (SI) and Relative Excess Risk Due To Interaction (RERI).

**Results:** For DDDs of NSAIDs upward of 0.50, a risk of UGIB was identified in carriers of the *3 allele (OR: 15,650, 95% CI: 1.41–174.10) and in carriers of the variant homozygous genotype (TT) of rs9923231 (OR: 38,850, 95% CI: 2.70–556.00). In LDA users, the risk of UGIB was observed to be similar between carriers of the wild type homozygous genotype and carriers of the variant alleles for the *CYP2C9* and *VKORC1* genes. No synergism was identified.

**Conclusion:** Our findings suggest an increased risk of UGIB in carriers of the variant allele of rs9923231 and in carriers of the *3 allele associated with doses of NSAIDs greater than 0.5. Hence, the assessment of these variants might reduce the incidence of NSAIDs-related UGIB and contribute to the safety of the NSAIDs user.

## Highlights

- This is the first full case-control study to assess the role of rs9923231 (*VKORC1* gene) in the risk of UGIB according to the genotype and the use of LDA and the DDD of NSAIDs consumed.
- This study is groundbreaking for evaluating *CYP2C9* alleles while considering not only the use of NSAIDs use but the use of LDA.
- Our findings suggest an increased risk of UGIB in carriers of the heterozygous (CT) and variant homozygous (TT) genotypes of rs9923231 (*VKORC1* gene) and in carriers of the *3 allele (rs10587910, *CYP2C9* gene) associated with daily doses of NSAIDs greater than 0.5.
- CYP2C9*3 allele might be used as a predictive marker of UGIB risk for *CYP2C9*-metabolized NSAIDs.
- Considering that NSAIDs are one of the most commonly prescribed classes in the world, these findings are of a high clinical interest because understanding individual genetic factors associated with UGIB risk might contribute to promoting patient safety and reducing harm associated with medication.

## Introduction

While several non-steroidal anti-inflammatory drugs (NSAIDs) are considered safe for over-the-counter use, especially when used short-term, they have the potential for serious adverse drug events, such as gastrointestinal toxicity and peptic ulcer (1). However, although the use of NSAIDs, including low-dose aspirin (LDA), is considered a risk factor for the development of peptic ulcer, only a few people develop peptic ulcer disease and its complications (e.g., perforated ulcer and upper gastrointestinal bleeding (UGIB)), which suggests idiosyncratic responses associated with possible individual risk factors or genetic susceptibility (2).

One hypothesis for idiosyncratic responses to the use of NSAIDs and LDA is the presence of variants in genes involved in the drug metabolism of these drugs or in physiological functions in the gastrointestinal tract system and platelet aggregation cascade (3). In a previous study we evaluated six variants in *PTGS1* and *NOS3* genes and an increased magnitude of UGIB risk was observed in NSAIDs and LDA users (4). In order to continue this investigation, we identified other genetic variants potentially involved in the risk of gastric injury and UGIB and the *2 (Arg144Cys, rs1799853) and *3 alleles (Ile359Leu, rs10587910) of *CYP2C9* gene stand out (3).

The gene encoding the cytochrome P-450 2C9 isoenzyme (*CYP2C9*) is highly polymorphic and it is responsible for metabolizing most NSAIDs, including aspirin, and drugs with a narrow therapeutic range (5). The presence of *CYP2C9* alleles may be associated with reduced isoenzyme activity (6) and several studies have investigated the relationship between the presence of these slow-metabolism variants and the risk of UGIB in warfarin users. Regarding the role of these variants in modifying the risk of UGIB in NSAIDs users, the data are inconsistent (7) and little is known about this risk in LDA users (3).

In warfarin users, the assessment of the *2 and *3 alleles of *CYP2C9* is often associated with the rs9923231 (-1639), located in a promoter region of the vitamin K epoxide reductase complex subunit 1 (*VKORC1* gene). Only one study assessed the influence of this variant in NSAIDs and LDA users and an increase of up to sevenfold in the risk of UGIB was identified in Romanians bearing the variant homozygous allele (8). However, the association data were reported grouping the use of LDA and NSAIDs; the dose of NSAIDs consumed was not considered, which prevents the gene-dose effect analysis; and it is not clear how drug exposure was measured. Besides, the authors did not assess whether the increase in UGIB risk is due either to the interaction between the genetic variant and NSAIDs and LDA use (synergism) or directly to the presence of the variant.

Along these lines, there is a lack of knowledge regarding the evidence linking genetic variation in *CYP2C9* and *VKORC1* genes to an increased rate of UGIB as an adverse drug event of NSAIDs and LDA use (1) and little is known about pharmacogenetic markers in the Latin American population (3). Hence, we intended to investigate whether interindividual variability in the *CYP2C9* and *VKORC1* genes is associated with increased risk of UGIB considering the use and gene-dose effect of LDA and NSAIDs, in addition to assessing the interaction or modification of effect between the genetic variants and LDA or NSAIDs intake on the risk of UGIB.

## Methods

### Study design, setting and ethical aspects

A full case-control study was conducted in the hospital complex of Clinical Hospital of the Ribeirão Preto Medical School of the University of São Paulo, Brazil, which comprises four hospitals that serve the population of the northwestern São Paulo sanitary region (49 municipalities and about 2,461,143 patients).

The Research Ethics Committee of the São Paulo State University (UNESP) (CAAE 53753115.4.3001.5426; protocol number 1.657.615) and Clinical Hospital of the Ribeirão Preto Medical School of the University of São Paulo (USP-RP) (CAAE 53753115.4.0000.5440; protocol number 1.536.886) approved the protocol of this study. Signed informed consent was obtained from all participants prior to their enrollment in this study.

The report of this study was based on Strengthening the Reporting of Observational Studies in Epidemiology (STROBE) (9).

### Definition of cases and control participants

Patients over 18 years old admitted to the hospital complex with signs and symptoms of UGIB and diagnosed by upper digestive endoscopy (UDE) or surgical intervention (laparoscopy) were determined as our study group.

UGIB was defined as: i) presence of endoscopically proven ulcers, perforation, or hemorrhagic erosions and ii) presence of dark or “coffee grounds” vomiting, melena, hematemesis, hematochezia, epidegastric pain, sudden loss, heavy sweating, and/or pallor.

Patient exclusion criteria were (i) bleeding from gastric or esophageal varices or neoplasm; (ii) presence of cirrhosis, *Mallory-Weiss* tears, and/or *Dieulafoy* lesions; (iii) serious health condition; (iv) UDE performed after 48 h of hospital admission; (v) hospitalization within 15 days prior to the current hospital admission; and (vi) in-hospital UGIB.

For each recruited case participant, controls were matched by sex, age (±5 years), and recruitment data (3 months). Control participants were admitted to preoperative units of the same hospital complex for mild surgery unrelated to gastrointestinal disorders (i.e., inguinal/umbilical hernia correction; plastic surgery; phacectomy (eye cataract); and prostatectomy).

Participants were recruited regardless of the use of NSAIDs and LDA in order to verify whether the proposed genetic variants are associated with the risk of UGIB or whether there is synergism between the variants and the use of these drugs in the risk of UGIB. Hence, it is essential that both case and control groups include NSAIDs or LDA-exposed and NSAIDs or LDA-unexposed individuals to assess the likely direct effect of functional variants on risk of suffering UGIB (7).

In order to reduce possible bias, only biologically unrelated participants were included. Participants with history of neoplasia, immunodeficiency syndrome, coagulopathies, nasogastric or percutaneous tube holders; patients who use narcotics; and non-residents of the study region for at least 3 months were excluded.

The inclusion and exclusion criteria for the participants are described in detail in our three previous studies (4, 10, 11).

### Data collection

Data collection was conducted face-to-face by four previously trained researchers (MF, GU, TRN and PCM), during the period from July 2016 to March 2020.

The interviews were carried out using a questionnaire previously designed for this study and validated to be applied in Brazil (12). The questionnaire comprises sociodemographic variables (sex; age; self-reported race (white, black, mixed, and East Asian); schooling); clinical (body mass index; previous personal history of gastrointestinal disorders (ulcer; dyspepsia; and bleeding); *Helicobacter pylori* infection; comorbidities; and drug therapy in use); and lifestyle habits (smoking, alcohol, and coffee intake).

The interviews were conducted with the patient and/or family member and the data were also consulted in secondary sources (electronic medical records, medical prescriptions, and laboratory tests). At the end of each interview, the researchers assigned a score from zero to 10 according to the quality of the information recalled (consistency of the interview).

After the interview, the venous blood (5 mL) was collected in ethylenediamine tetraacetic acid (EDTA) from all participants for posterior analysis of the proposed genetic variants and serology for *Helicobacter pylori* infection.

### Definition of drug exposure and lifestyle

An index date was considered, which is deemed for each case as the day of appearance of dark vomiting or in coffee grounds, melena, and/or hematemesis (signs and symptoms of UGIB) and for controls the index date was defined as the date of the interview (13). To assess an association between the use of NSAIDs and LDA with the risk of UGIB, a 7-day etiologic window dated from the index date was considered, in line with other published studies (12, 14).

For *CYP2C9* analysis, NSAIDs use was grouped into NSAIDs metabolized at least 50% by *CYP2C9* (piroxicam, celecoxib, naproxen, aceclofenac, indomethacin, diclofenac, and ibuprofen) and into NSAIDs metabolized less than 50% by *CYP2C9* (other NSAIDs) (12). It is known that NSAIDs, as they are substrates of *CYP2C9*, are not metabolized at the same proportion of this enzyme and to perform an analysis considering the proportion of each NSAIDs metabolized by *CYP2C9* increases the sample size power (7). LDA use was deemed the continuous use of aspirin at doses below 300 mg per day in the indication of prevention of primary and secondary cardiovascular events (15).

To perform an analysis of dose-effect, two researchers calculated the defined daily dose (DDD) by the World Health Organization (WHO) for NSAIDs for all participants. DDD was defined as the average maintenance dose per day of a drug used for its main indication in adults in the 7-day etiologic window preceding the data index. The dose-response effect was assessed using three categories: NSAIDs non-users (NSAIDs DDD = 0), NSAIDs users of 0.50 DDD or less (>0 NSAIDs DDD ≤0.50), and NSAIDs users of over 0.50 DDD (NSAIDs DDD >0.50) based on the proposal by Figueiras et al. (12). This approach considered each type of NSAIDs and the recommended DDD, enabling different NSAIDs to be compared with one another (7).

Regarding lifestyle, the mean daily consumption of tobacco, alcohol, and coffee over the 2 months preceding the index was calculated. Smoking habit was stratified according to the number of cigarettes consumed per day: non-smokers and ex-smokers (zero cigarette); 1 to 15 cigarettes/day; and >15 cigarettes/day. Alcohol intake was stratified in abstainers (0 g), >0 to ≤30 g of alcohol/day, and >30 g of alcohol. Coffee intake was stratified into none consumed (0 mL), >0 mL ≤ 100, >100 > mL ≤ 300, and >300 mL.

### *Helicobacter pylori* serology

For all the participants, the presence of IgG antibodies for *Helicobacter pylori* infection (anti-*H. pylori* IgG antibodies) was determined using the chemiluminescence technique. This parameter was stratified into reagent (≥1.10 U IgG/mL), non-reagent (≤0.90 U IgG/mL), and inconclusive (0.91–1.09 U IgG/mL). In addition, all participants were queried about previous *Helicobacter pylori* infection.

### Selection of genetic variants and genotyping

The genetic variants proposed in this study were selected through a systematic review conducted by our research group (3). The single nucleotide polymorphisms *CYP2C9* Arg144Cys (rs1799853, A > C; Assay C_25625805_10); *CYP2C9* Ile359Leu (rs10587910, C > T, Assay C_27104892_10); and rs9923231 (−1,639, C > T, Assay C_30403261_20) were evaluated.

Genomic DNA was extracted from venous blood samples using the Maxwell® 16 Blood DNA Purification Kit (Promega, Madison, WI, United States). DNA concentration and purity were determined by fluorescence technique using the Invitrogen Qubit 4 Fluorometer and the Qubit ™ dsDNA HS Assay kit (Applied Biosystems, Foster City, United States).

Genetic variants genotyping was performed on genomic DNA by real-time polymerase chain reaction technique using the TaqPath ProAmp SNP Genotyping Assay system (Applied Biosystems, Foster City, United States) and StepOne Plus equipment (Applied Biosystems, Foster City, United States).

The cycling conditions were validated as 60°C for 1 min; 95°C for 10 min; 40 cycles of 95°C for 15 s and 60°C for 1 min; and final extension of 60°C for 1 min.

For internal quality control, for each of the variants, 10% of the samples were randomly selected for repeat genotyping (90 samples per variant). The agreement between the two sets of genotyping was 100%.

Genotyping plots were analyzed using the Data Connect cloud from Applied Biosystems (Foster City, United States).

### Statistical analysis

Intergroup comparison for qualitative variables was calculated using the χ2 test or Fisher’s exact test, if appropriate, and Student's t-test for quantitative variables. The difference between the allele frequency of the genetic variants between the case and control groups was tested with χ2 test.

#### *Hardy-Weinberg* Equilibrium

The genotype distributions of the genetic variants proposed were verified in the control group conforming to *Hardy–Weinberg* Equilibrium using χ2 test (BioEstat 5.0 program).

#### Unconditional regression models

The association between the *CYP2C9* and *VKORC1* variant alleles and the risk to develop UGIB was estimated by odds ratio (OR) with 95% confidence interval (95% CI) using unconditional logistic regression models (SPSS v.26 software (IBM Company, Chicago, IL, United States)).

Firstly, a bivariate analysis was carried out to test the effect of the potentially confounding variables. Variables with *p*-value ≤0.10 were selected to compose the unconditional logistic regression models. The variables sex and age were not included in the models, since they were controlled in the study design by matching the participants of the case and control groups.

The unconditional logistic regression model was built with all confounding variables selected in the bivariate analysis (insertion method of the SPSS Program).

To evaluate the *CYP2C9* alleles, participants were grouped in carriers of CYP2C9*1, CYP2C9*2 and CYP2C9*3 alleles and in carriers of genotypes without *3 allele (*1/*1; *1/*2, or *2/*2) and carriers with *3 allele (*1/*3; *3/*3, or *2/*3). For the *VKORC1* alleles, the participants were grouped in carriers of wild type homozygous, heterozygous, and variant homozygous genotypes. Both groups considered the dose of NSAIDs consumed or LDA use.

#### Multiplicative and additive scale interaction

For interaction analysis, the participants were divided into four groups according to genotype and NSAIDs/LDA use status, being:

00: absence of genetic variation and no use of NSAIDs and/or LDA
01: absence of genetic variation and use of NSAIDs and/or LDA
10: genetic variation and no use of NSAIDs and/or LDA
11: genetic variation and use of NSAIDs and/or LDA

Multiplicative interactions between the presence of the *CYP2C9* and *VKORC1* alleles and the use of NSAIDs or LDA was calculated by fitting the unconditional regression models.

The additive interaction between the presence of the *CYP2C9* and *VKORC1* alleles and the use of NSAIDs or LDA was estimated by employing the Synergy Index (SI) and Relative Excess Risk Due to Interaction (RERI) parameters (16) as proposed by Anderson and collaborators (17). 95%CI was constructed according to the method proposed by Figueiras et al. (18).

$$SI=\left({OR}_{11}\;–\;1\right)/\left(\left({OR}_{01}\;–\;1\right)+\left({OR}_{10}\;–\;1\right)\right)$$

$$RERI={OR}_{11}\;–\;{OR}_{01}\;–\;{OR}_{10}+1$$

SI > 1 or RERI >0 indicated a significant additive interaction, which represents that the combined effects of the presence of the variant alleles and exposure to NSAIDs or LDA on UGIB risk are greater than the effects of drug exposure or presence of variant alleles alone.

Regression models outlined for multiplicative and additive interactions analysis were fitted for the selected confounding variables in the bivariate analysis (*p*-value ≤0.10).

#### Model fit measures

To avoid distortions of the model effect measures, the potential problem of multicollinearity between independent variables was evaluated based on correlation matrix of regression coefficients.

Four methods were used to measure the goodness and quality of the statistical models’ fit: Akaike information criterion (AIC), Bayesian information criteria (BIC), Hosmer and Lemeshow, and likelihood ratio test (2LL) (2LL block zero – 2LL in block one). A low AIC and BIC scores indicate a better model fit for predicting outcome.

## Results

### Baseline characteristics of case and Control Participants

Most participants were male (case group: 72.5% and control group: 72.5%), self-declared white (case group: 81.3% and control group: 84.2%), with zero to 4 years of schooling (case group: 52.5% and control group: 52.4%), and with a mean age of 60.0 (±15.9) for case group and 59.9 (±15.9) years for control group. The demographic and clinical characteristics of the participants are described in Table 1.

**TABLE 1:** Demographic and clinical characteristics of the participants of case (*n* = 200) and control (*n* = 706) groups.

	Case (%) *n* = 200	Control (%) *n* = 706	*p*-value^a^
Demographic variables			
Sex (male)	145 (72.5)	512 (72.5)	0.933
Age [mean (±SD)]	60.2 (16.3)	59.8 (15.8)	0.750
Race (self-declared)			0.406
White	143 (81.3)	542 (84.2)	
mixed	33 (18.8)	102 (15.8)	
Black	23 (11.5)	58 (8.2)	
East Asian	1 (0.5)	4 (0.6)	
Interview consistency			0.002^b^
Scores 6 and 7	91 (45.5)	223 (31.6)	
Scores 8 and 9	77 (38.5)	324 (45.9)	
Score 10	32 (16.0)	159 (22.5)	
Body mass index kg/m^2^			<0.001^b^
Underweight (<18)	10 (5.0)	15 (2.1)	
Normal (≥18 - ≤ 24)	80 (40.0)	197 (27.9)	
Overweight (≥25 - ≤ 29.9)	60 (30.0)	263 (37.3)	
Obesity (≥30)	48 (24.0)	228 (32.3)	
Missing data	2 (1.0)	3 (0.4)	
Personal history of gastrointestinal diseases			
History of ulcer	44 (22.1)	64 (9.1)	<0.001^b^
History of bleeding	35 (17.6)	94 (13.3)	0.159
History of dyspepsia	60 (30.2)	291 (41.2)	0.006^b^
*Helicobacter pylori* infection			
*Helicobacter pylori* infection (IgG test)	142 (76.3)	388 (57.6)	<0.001^b^
*Helicobacter pylori* infection (self-report)	12 (6.0)	50 (7.0)	0.706
Comorbidity			
Cardiovascular disease	62 (31.0)	133 (18.8)	<0.001^b^
High blood pressure	104 (52.0)	371 (52.5)	0.954
Diabetes *mellitus*	38 (19.1)	158 (22.4)	0.370
Dyslipidemia	22 (11.0)	165 (23.4)	0.001^b^
Depression	20 (10.1)	81 (11.5)	0.663
Arthrosis	9 (4.5)	48 (6.9)	0.296
Arthritis	3 (1.5)	22 (3.1)	0.364
Drug therapy in use (ATC)			
Proton pump inhibitors (A02BC)	36 (18.0)	125 (17.7)	0.993
Oral anticoagulants (B01A)	22 (11.0)	18 (2.5)	<0.001^b^
LDA (B01AC06)	51 (25.5)	90 (12.7)	<0.001^b^
Other antiplatelet agents (B01AC)	26 (13.0)	61 (8.6)	0.065^b^
NSAIDs metabolized by *CYP2C9*			<0.001^b^
DDDs of NSAIDs = 0	171 (85.5)	656 (92.9)	
>0 DDDs of NSAIDs ≤0.50	19 (9.5)	41 (5.8)	
DDDs of NSAIDs >0.50	10 (5.0)	9 (1.3)	
Tobacco consumption			<0.001^b^
Non-smoker/ex-smoker (0 cigarette)	136 (68.0)	580 (82.2)	
1 to 15 cigarettes/day	23 (11.5)	50 (7.0)	
>15 cigarettes/day	35 (17.5)	55 (7.8)	
Missing data	6 (3.0)	21 (3.0)	
Alcohol intake			<0.001^b^
Abstainer (0 g)	103 (51.5)	392 (55.5)	
0 to ≤30 g of alcohol/day	71 (35.5)	297 (42.1)	
>30 g of alcohol/day	26 (13.0)	17 (3.3)	
Coffee intake			0.011^b^
mL = 0	30 (15.0)	66 (9.2)	
>0 mL ≤ 100	13 (6.5)	39 (5.5)	
>100 > mL ≤ 300	124 (62.0)	520 (73.6)	
>300 mL	33 (16.5)	82 (11.6)	

^a^ *p*-value is polychotomous and represents the entire variable.

^b^ variables with *p*-value ≤0.10 and selected for the unconditional regression models.

ATC, anatomical therapeutic chemical; DDD, defined daily dose; IgG, Immunoglobulin G; LDA, low-dose aspirin; mL, milliliter; NSAIDs, non-steroidal anti-inflammatory drugs; OR, odds ratio; SD, standard deviation.

As indicated in Table 1, participants in the case group received more NSAIDs and LDA (21.5% and 25.5%) when compared with the participants of the control group (10.0% and 12.7%). Concomitant use of NSAIDs and LDA was reported by 4.0% of cases and 1.1% of controls. 14.5% of the participants in the case group were using NSAIDs metabolized by *CYP2C9* and 7.0% of the participants in the control group, and the most commonly used NSAIDs were diclofenac and nimesulide. In relation to DDD, 2.5% of cases and 0.8% of controls used NSAIDs in DDD of 1.0 upward.

The three variants were in accordance with the *Hardy-Weinberg* Equilibrium: CYP2C9*2 (*p*-value 0.6773 for the case group and 0.7144 for the control group), CYP2C9*3 (*p*-value 0.9492 for the case group and 0.9570 for the control group), and *VKORC1* (*p*-value 0.5407 for the case group and 0.5076 for the control group). No differences were observed between the frequency of *CYP2C9* and *VKORC1* genotypes in the case and control groups (Table 2).

**TABLE 2:** Frequency of *CYP2C9* and *VKORC1* genotypes among participants in the case (*n* = 200) and control (*n* = 706) groups.

	Case N (%)	Control N (%)	*p*-value
Gene *CYP2C9*			
CYP2C9*2 (Arg144Cys)			0.935
*CYP2C9* 144Arg/Arg	165 (82.5)	583 (82.5)	
*CYP2C9* 144Arg/Cys	32 (16.0)	118 (16.7)	
*CYP2C9* 144Cys/Cys	1 (0.5)	5 (0.7)	
Missing data	2 (1.0)	0	
CYP2C9*3 (Ile359Leu)			0.383
*CYP2C9* 359Ile/Ile	161 (80.5)	542 (76.8)	
*CYP2C9* 359Ile/Leu	35 (17.5)	153 (21.7)	
*CYP2C9* 359Leu/Leu	2 (1.0)	11 (1.5)	
Missing data	2 (1.0)	0	
Combined CYP2C9*2 (Arg144Cys) and CYP2C9*3 (Ile359Leu) genotypes			0.417
Wild type homozygous (*1/*1)	131 (65.5)	441 (62.5)	
Heterozygous (*1/*2 or *1/*3)	61 (30.5)	230 (32.6)	
Variant homozygous or compound heterozygous (*2/*2 or *3/*3 or *2/*3)	6 (3.0)	35 (4.9)	
Missing data	2 (1.0)	0	
Gene *VKORC1*			
rs9923231 (−1,639, C > T)			0.298
CC (wild type homozygous)	86 (43.0)	265 (37.5)	
CT (heterozygous)	86 (43.0)	327 (46.3)	
TT (variant homozygous)	26 (13.0)	112 (15.8)	
Missing data	2 (1.0)	3 (0.4)	

N: number of participants of case and control group.

C > T: represents the base exchange of cytosine for thymine.

### Association of genetic variants and the risk of UGIB

Risk of UGIB associated with each allele was analyzed taking the NSAIDs dose consumed into account (Table 3).

**TABLE 3:** Risk for upper gastrointestinal bleeding associated with *CYP2C9* and *VKORC1* genotypes and dose of non-steroidal anti-inflammatory drugs (NSAIDs) consumed.

	N (case/control)	OR^a^	95%CI^a^	*p*-value^a^
Gene *CYP2C9*				
Allele CYP2C9*1				
DDDs of NSAIDs = 0 (Ref)	101/406	1.000		
>0 DDDs of NSAIDs ≤0.50	11/24	4.871	1.98–11.96	0.001*
DDDs of NSAIDs >0.50	6/6	6.676	1.79–24.90	0.005*
Allele CYP2C9*2				
DDDs of NSAIDs = 0	18/91	0.581	0.28–1.19	0.138
>0 DDDs of NSAIDs ≤0.50	7/7	6.244	1.56–24.92	0.009*
DDDs of NSAIDs >0.50	1/1	10.618	0.61–186.17	0.106
Allele CYP2C9*3				
DDDs of NSAIDs = 0	33/147	1.189	0.70–2.01	0.519
>0 DDDs of NSAIDs ≤0.50	0/10	-	-	-
DDDs of NSAIDs >0.50	3/2	15.650	1.41–174.10	0.025*
Genotypes without CYP2C9*3				
DDDs of NSAIDs = 0 (Ref)	119/497	1.000		
>0 DDDs of NSAIDs ≤0.50	18/31	5.217	2.44–11.14	<0.001*
DDDs of NSAIDs >0.50	7/7	7.368	2.19–24.74	0.001*
Genotypes with CYP2C9*3				
DDDs of NSAIDs = 0	33/147	0.956	0.55–1.65	0.874
>0 DDDs of NSAIDs ≤0.50	0/10	-	-	-
DDDs of NSAIDs >0.50	3/2	15.086	1.34–169.66	0.028*
Missing data	20/12			
Gene *VKORC1* rs9923231 (-1,639, C > T)^b^				
CC (wild-type homozygous)				
DDDs of NSAIDs = 0 (Ref)	66/239	1.000		
>0 DDDs of NSAIDs ≤0.50	70/295	0.922	0.57–1.47	0.735
DDDs of NSAIDs >0.50	21/99	0.788	0.41–1.51	0.475
CT (heterozygous)				
DDDs of NSAIDs = 0	11/20	2.259	0.81–6.27	0.118
>0 DDDs of NSAIDs ≤0.50	13/24	6.025	2.40–15.12	<0.001*
DDDs of NSAIDs >0.50	3/12	3.142	0.71–13.77	0.129
TT (variant homozygous)				
DDDs of NSAIDs = 0	9/6	8.801	2.41–32.01	0.001*
>0 DDDs of NSAIDs ≤0.50	3/8	2.778	0.64–11.92	0.169
DDDs of NSAIDs >0.50	2/1	38.850	2.70–556.0	0.007*
Missing data	2/2			

^a^ Analysis adjusted for the following confounding variables: body mass index kg/m^2^; personal history of ulcer and dyspepsia; *Helicobacter pylori* infection; cardiovascular disease; dyslipidemia; use of oral anticoagulants, LDA, other antiplatelet agents; tobacco consumption; alcohol intake; coffee intake; and interview consistency.

^b^ For the analysis of rs9923231considering the consumption of NSAIDs, there was no restriction for NSAIDs metabolized by *CYP2C9*. Therefore, use and non-use of NSAIDs were considered, regardless of the metabolism pathway.

- Lack of association data (OR, 95% CI and *p*-value) due to impossibility of performing statistical analysis for these categories, considering that no participant in the case group carried the variant allele.

Genotypes without CYP2C9*3 (*1/*1; *1/*2, or *2/*2).

Genotypes with CYP2C9*3 (*1/*3; *3/*3, or *2/*3).

C > T: represents the base exchange of cytosine for thymine.

95% CI: confidence interval; DDD, defined daily dose; **N**, number of participants of case and control group; NSAIDs, Non-steroidal anti-inflammatory drugs; OR, odds ratio; Ref, reference category.

For DDDs of NSAIDs up to 0.50, the risk of UGIB was similar for wild type homozygous (OR: 4.871, 95% CI: 1.98–11.96) and carriers of the *2 allele (OR: 6.244, 95% CI 1.56–24.80). It was not possible to evaluate UGIB risk among carriers of the *3 allele in use of DDDs of NSAIDs up to 0.50, as there was no case in this category. For DDDs of NSAIDs upward 0.50, the risk of UGIB was much lower for the wild type homozygous (OR: 6.676, 95% CI: 1.79–24.90) when compared with carriers of the *3 allele (OR: 15.650, 95% CI: 1.41–174.10). From DDDs of NSAIDs upward 0.50, a greater risk of UGIB was observed among carriers of CYP2C9*3 genotypes (15.086, 95% CI 1.34–169.66) when compared to carriers of genotypes with *2 allele and/or wild type homozygous (OR: 7.368, 95% CI: 2.19–24.74).

For the *VKORC1* gene, a higher risk of UGIB was observed in carriers of the variant homozygous genotype (TT) in use of DDDs of NSAIDs upward of 0.50 (OR: 38.850, 95% CI: 2.70–556.00) when compared to carriers of the heterozygous genotype (CT) (OR: 6.025, 95% CI: 2.40–15.12).

In LDA users, the risk of UGIB was observed to be similar between carriers of the wild type homozygous genotype and carriers of the variant alleles for the *CYP2C9* and *VKORC1* genes (Table 4).

**TABLE 4:** Risk for upper gastrointestinal bleeding associated with *CYP2C9* and *VKORC1* genotypes and use of low-dose aspirin (LDA).

	N (case/control)	OR	95% CI	*p*-value
Gene *CYP2C9*				
Allele CYP2C9*1				
LDA use (no) (Ref)	97/387	1.000		
LDA use (yes)	34/54	3.692	1.76–7.76	0.001*
Allele CYP2C9*2				
LDA use (no)	22/88	0.778	0.39–1.53	0.471
LDA use (yes)	7/12	1.664	0.39–7.10	0.492
Allele CYP2C9*3				
LDA use (no)	28/141	1.107	0.70–2.01	0.519
LDA use (yes)	10/24	2.911	1.01–8.45	0.050
Genotypes without CYP2C9*3				
LDA use (no)	119/475	1.000		
LDA use (yes)	41/66	3.441	1.79–6.61	<0.001*
Genotypes with CYP2C9*3				
LDA use (no)	25/141	1.148	0.66–2.00	0.628
LDA use (yes)	10/24	3.055	1.14–8.21	<0.001*
Missing data (*CYP2C9* gene)	2/0			
Gene *VKORC1* rs9923231 (-1639, C > T)				
CC (wild type homozygous)				
LDA use (no)	57/229	1.000		
LDA use (yes)	29/36	4.017	1.17–9.40	0.001*
CT (heterozygous)				
LDA use (no)	69/287	1.033	0.62–1.71	0.901
LDA use (yes)	17/40	3.473	1.37–8.79	0.009*
TT (variant homozygous)				
LDA use (no)	21/98	1.093	0.56–2.14	0.796
LDA use (yes)	5/14	1.131	0.19–6.66	0.891
Missing data (*VKORC1* gene)	2/2			

Genotypes without CYP2C9*3 (*1/*1; *1/*2, or *2/*2).

Genotypes with CYP2C9*3 (*1/*3; *3/*3, or *2/*3).

C > T: represents the exchange of cytosine bases for thymine.

95% CI: confidence interval; DDD, defined daily dose; LDA, low-dose aspirin; N, number of participants of case and control group; OR, odds ratio; Ref, reference category. Analysis adjusted for the following confounding variables: body mass index kg/m^2^; personal history of ulcer and dyspepsia; *Helicobacter pylori* infection; cardiovascular disease; dyslipidemia; use of oral anticoagulants, other antiplatelet agents, NSAIDs; tobacco consumption; alcohol intake; coffee intake; and interview consistency.

All these models were adjusted for the following confounding variables: body mass index kg/m^2^; personal history of ulcer and dyspepsia; *Helicobacter pylori* infection; cardiovascular disease; dyslipidemia; use of oral anticoagulants, other antiplatelet agents, NSAIDs drugs; tobacco consumption; alcohol intake; coffee intake; and interview consistency.

No additive interaction was identified between the presence of variant alleles of the *CYP2C9* and *VKORC1* genes and the consumption of NSAIDs or LDA. Although not statistically significant, the absence of the CYP2C9*3 genotype (RERI: −2.17, CI 95%: −5.16; 0.82) and rs9923231 variant (*VKORC1*) (RERI: −1.23, CI 95%: −4.49; 2.03) appear to be “negative” modifiers for the risk of UGIB in LDA users (Table 5).

**TABLE 5:** Multiplicative and additive interaction between the presence of the *CYP2C9* e *VKORC1* alleles and the use of low-dose aspirin (LDA) and non-steroidal anti-inflammatory drugs (NSAIDs) in the risk of upper gastrointestinal bleeding.

	Wild type		*p*-value	Genetic variation		*p*-value	RERI (95% CI)	SI (95% CI)
	N/N case/control	OR (95% CI)		N/N case/control	OR (95% CI)			
Gene *CYP2C9*								
LDA								
*CYP2C9* without CYP2C9*3 genotype								
No use	97/387	1.00 (References)		22/88	0.78 (0.39–1.54)	0.471	−2.17 (−5.16; 0.82)	0.12 (0.00; 53.28)
Use	34/54	3.69 (1.76–7.76)	<0.001^a^	7/12	1.66 (0.39–7.11)	0.492		
*CYP2C9* with CYP2C9*3 genotype								
No use	-	-	-	28/141	1.11 (0.63–0.95)	0.725	−0.89 (−6.99; 5.22)	0.68 (0.03; 14.99)
Use	-	-	-	10/24	2.91 (1.00–8.46)	0.050^a^		
NSAIDs								
*CYP2C9* without CYP2C9*3 genotype								
No use	101/406	1.00 (References)		18/91	0.59 (0.29–1.20)	0.146	−3.80 (−8.01; 0.41)	0.12 (0.00; 53.28)
Use	17/30	5.31 (2.48–11.35)	<0.001^a^	8/8	6.89 (1.96–24.21)	0.003^a^		
*CYP2C9* with CYP2C9*3 genotype								
No use	-	-	-	33/143	1.19 (0.70–2.01)	0.518	−0.89 (−6.99; 5.22)	0.68 (0.03; 14.99)
Use	-	-	-	3/12	1.38 (0.26–6.73)	0.688		
Gene *VKORC1* (rs9923231, -1,639, C > T)								
LDA								
No use	57/229	1.00 (References)		90/385	1.04 (0.65–1.68)	0.850	−1.23 (−4.49; 2.03)	0.60 (0.16; 2.21)
Use	36/29	4.01 (1.72–9.38)	0.001^a^	22/54	2.83 (1.17–6.83)	0.021^a^		
NSAIDs								
No use	59/237	1.00 (References)		80/384	0.89 (0.56–1.41)	0.615	1.54 (−3.23; 6.32)	1.48 (0.43; 5.14)
Use	20/26	4.32 (1.90–9.80)	<0.001^a^	21/45	5.74 (2.74–12.03)	<0.001^a^		

^a^ Statistical significance.

- Whereas the variable “*CYP2C9* with CYP2C9*3 genotype” represents participants carrying the CYP2C9*3 genotype, there are no participants carrying the wild type.

C > T: represents the exchange of cytosine bases for thymine.

Analysis adjusted for the following confounding variables: body mass index kg/m^2^; personal history of ulcer and dyspepsia; *Helicobacter pylori* infection; cardiovascular disease; dyslipidemia; use of oral anticoagulants, other antiplatelet agents, NSAIDs (in LDA models), LDA (in NSAIDs models); tobacco consumption; alcohol intake; coffee intake; and interview consistency.

95% CI, confidence interval; LDA, low-dose aspirin; N, number of participants of case and control group; NSAIDs, Non-steroidal anti-inflammatory drugs; OR, odds ratio; RERI, Relative Excess Risk Due to Interaction; SI, Synergism Index.

Model fit measures of RERI and SI for LDA users: -2LL: 610.310; AIC: 658.310; BIC: 677.206; Hosmer and Lemeshow: 0.726.

Model fit measures of RERI and SI for NSAIDs users: -2LL: 610.327; AIC: 630.327; BIC: 677.223; Hosmer and Lemeshow: 0.726.

All these models were adjusted for the following confounding variables: body mass index kg/m^2^; personal history of ulcer and dyspepsia; *Helicobacter pylori infection*; cardiovascular disease; dyslipidemia; use of oral anticoagulants, LDA, other antiplatelet agents, and NSAIDs; tobacco consumption; alcohol intake; coffee intake; and interview consistency.

## Discussion

To the best of our knowledge, this is the first full case-control study to assess the role of rs9923231 (*VKORC1* gene) in the risk of UGIB according to the genotype and the use of LDA and DDD of NSAIDs consumed. To consider the dose of NSAIDs is essential, since there is evidence of the presence of gene-dose effect and dose-dependency in NSAIDs-induced gastrointestinal damage (7, 12). Additionally, this study is groundbreaking for evaluating *CYP2C9* alleles considering not only the use of NSAIDs use but the use of LDA and to explore additive synergism interaction between the *VKORC1* and *CYP2C9* variants and NSAIDs/LDA on the UGIB risk (RERI and SI).

Our findings suggest an increased risk of UGIB in carriers of the heterozygous (CT) and variant homozygous (TT) genotypes of rs9923231 (*VKORC1* gene) and in carriers of the *3 allele (rs10587910, *CYP2C9* gene) associated with increased dose of NSAIDs consumed. Considering that NSAIDs are one of the most commonly prescribed classes in the world, the limited understanding of the molecular mechanisms involved in idiosyncratic responses (1), and the public health and economic burden of UGIB (2), these findings are of a high clinical interest because knowing individual genetic factors associated with UGIB risk may contribute to promoting patient safety and reducing the medication harm.

The *VKORC1* gene encodes the vitamin K epoxide reductase protein, a key enzyme in the vitamin K cycle (19), and the rs9923231 is one of the most studied variants. It is suggested that this variant is associated with a low-dose phenotype and changes in the transcription factor binding site of the gene (20), and luciferase assays show that the activity of the wild type allele of rs9923231 is increased by 44% over the activity of the homozygous variant allele (20). In this respect, one hypothesis for the increased risk of UGIB observed in carriers of the variant allele of rs9923231 would be this variant, located in a promoter region, which might lead to reduced promoter activity and, consequently, reduced levels of functional clotting factors and an increased likelihood of bleeding (8).

A previous study investigated whether the rs9923231 would be involved in the increased risk of UGIB in LDA and NSAID users (8), since this variant is widely evaluated in vitamin K antagonist users, as it is associated with increased sensitivity to warfarin and a low dose requirement to prevent bleeding episodes (21). The authors included 341 participants and identified about seven-fold increased risk in carriers of the variant homozygous genotype using NSAIDs or LDA (OR: 3.12–18.74) (8), whereas in our study we included 906 participants and only the users of NSAIDs in DDDs upward 0.50 bearing the variant homozygous genotype had an increased risk of UGIB (OR: 38.850, 95% CI: 2.70–556.00). It is well known that the risk of NSAID-induced gastrointestinal complications is dose-dependent and remains linear over time (22). Therefore, it is tough to compare the authors’ data with our findings, as the use of LDA and NSAIDs was grouped and the dose of NSAIDs consumed was not considered. In addition, in our study users of LDA carrying the variant allele did not present a higher risk of UGIB.

Regarding *CYP2C9*, variants in this gene may result in modified expression or functionality, correlating with altered drug metabolism and clearance, which may affect drug bioavailability (23) and it was suggested that the “decreased-function” and “no-function” *CYP2C9* alleles are substrate-dependent (22). Indeed, a reduction in *CYP2C9* activity of about 20–30% was observed in carriers of the *2 allele and up to 70% in carriers of the *3 allele (6), in addition to variations in NSAIDs metabolism as well as NSAID-induced adverse drug reactions (22, 24).

We are aware of only one previous full case-control study that evaluated the influence of *CYP2C9* alleles and dose of NSAIDs consumed on UGIB risk (12), since previous studies were based on NSAIDs users (partial case control) (25, 26), which prevents assessing whether the risk of UGIB is directly related to the presence of a functional variant or interaction between slow-metabolizing variants and use of NSAIDs (7).

Figueiras et al. (2016) identified that carriers of the allele *3 users of NSAIDs in DDDs greater than 0.5 have a higher risk of UGIB (OR: 16.920, 95% CI: 4.96–57.59) (12), corroborating with our findings (OR: 15.650, 95% CI: 1.41–174.10). This finding may suggest the presence of a gene-dose effect and the risk and severity of NSAIDs toxicity are likely to be increased in individuals with a poor metabolizer phenotype of *CYP2C9* because they are expected to exhibit a pronounced prolongation of drug half-life and increase in plasmatic concentration (27, 28). In line with previous studies, the *2 allele was not associated with an increased risk of UGIB (12, 25) and this allele was reported as a poor risk predictor of gastrointestinal adverse events (24).

From a practical point of view, thinking about the safety of the NSAIDs user, therapeutic recommendations involve dose reduction or alternative therapies, including NSAIDs not primarily metabolized by *CYP2C9* or with pharmacokinetic not impacted by *CYP2C9* genetic variants (1, 23), coupled with careful monitoring of adverse drug events (1). The care of NSAIDs users should be extended even further in the older person, considering that multimorbidity and polypharmacy increase the likelihood of drug and gene-based interactions (29). Besides, the older person is likely to be prescribed NSAIDs (29, 30) and this pharmacological class is considered inappropriate for older people (31).

Regarding the use of LDA, it was not identified modification of the magnitude of risk of UGIB in individuals bearing the *2 and *3 alleles (*CYP2C9* gene) or the variant allele of rs9923231 (*VKORC1* gene) and the pharmacokinetics of LDA does not seem to be significantly affected by these genetic variants (1). In contrast to NSAIDs, CYP metabolism may only play a minor role in LDA bioavailability and no strong genetic associations with aspirin-related gastrointestinal events have been described so far (32, 33).

Notwithstanding, our data corroborates evidence on the pharmacogenomics of LDA, which is less explored in Brazilian studies (21), despite UGIB being the most commom adverse drug event observerd in 72% of aspirin-related hospitalizations (34). In addition, gastrointestinal symptoms in chronic LDA users are challenging and are a result of non/poor compliance and discontinuation of treatment (35), which may be associated with an increased risk of new cardiovascular events (36).

Ultimately, although it is known that the adoption of Pharmacogenetics-guided prescribing in Brazil has been sporadic due to a combination of factors, such as the structure of the Brazilian population and barriers to its implementation (37), genetic data is a useful tool to improve pharmacotherapy, reduce the occurrence of adverse drug reactions, and change the paradigm of dosing regimens being extrapolated to entire populations (38, 39). Thus, our findings may contribute to personalized therapy and to improve the detection of the NSAIDs-related UGIB signal, since improving risk communication and signal detection is one of the demands of Brazilian Pharmacovigilance (40).

Our study has strength and limitations. This is the first time this type of study was performed to explore pharmacogenomics associations in individuals with admixed genetic ancestry in addition to collecting data through face-to-face interviews and adjusting the analyses for several UGIB confounders. The main limitation of this study is the sample size, because the stratification of participants according to genotype and DDD of NSAIDs consumed meant that some categories had a limited number of observations, which reduces statistical power and is a frequent limitation in genetic studies (41). Furthermore, additional studies including analyzes of ancestry-informative markers would be relevant, as there are differences in the distribution of variants in the *CYP2C9* and *VKORC1* genes, especially as the proportion of African ancestry increases (35).

In summary, in this exploratory study, our findings suggest an increased risk of UGIB in carriers of the heterozygous (CT) and variant homozygous (TT) genotypes of rs9923231 (*VKORC1* gene) and in carriers of the *3 allele (*CYP2C9* gene) associated with increased dose of NSAIDs consumed. Besides, no additive synergism interaction was identified and the joint biological effect of these variants and NSAIDs/LDA use on the risk of UGIB is similar to the sum of their individual effects.

Hence, our findings may contribute to personalized pharmacotherapy and to improve the detection of the NSAIDs-related adverse drug reactions.

## Data Availability

The raw data supporting the conclusion of this article will be made available by the authors, without undue reservation, to any qualified researcher. Besides, the full-methodology protocol, interview form is available at Open Science Framework (OSF), an open access repository (doi: 10.17605/OSF.IO/4SG93).
